# Extended planar defects of oxygen vacancies in ferroelectric $\hbox {BaTiO}_3$ and impact on ferroelectricity

**DOI:** 10.1038/s41598-023-46489-y

**Published:** 2023-11-10

**Authors:** Shaohui Qiu, Huaxiang Fu

**Affiliations:** 1 Department of Physics, University of Arkansas, Fayetteville, AR 72701 USA; 2 Department of Chemistry and Physics, Southern Utah University, Cedar City, UT 84720 USA

**Keywords:** Materials science, Physics

## Abstract

Extended defects of vacancies in ferroelectrics (FE), where vacancies spread over an extended space, are of critical importance in terms of understanding the long-standing problems such as polarization fatigue and aging. However, extended defects in FEs are poorly understood. Here we investigate the extended planar oxygen vacancies in ferroelectric $\hbox {BaTiO}_3$ using density functional theory and the modern theory of polarization. Oxygen vacancies of different charge states, namely ${\textrm{V}}^{2+}_{{\textrm{O}}}$, ${\textrm{V}}^{1+}_{{\textrm{O}}}$, and ${\textrm{V}}^{0}_{{\textrm{O}}}$, are studied. We obtain interesting results such as: (i) The formation energy of planar ${\textrm{V}}^{2+}_{{\textrm{O}}}$ vacancies can be very small (merely 0.54 eV) even under the oxygen-rich condition, which is considerably smaller than the formation energy (4.0 eV) of planar ${\textrm{V}}^{0}_{{\textrm{O}}}$ vacancies; (ii) Planar ${\textrm{V}}^{2+}_{{\textrm{O}}}$ vacancies drastically reduce the ferroelectric polarization in $\hbox {BaTiO}_3$ by more than one order of magnitude, which provides a pivotal (theoretical) evidence that the planar oxygen vacancies could be the origin of polarization fatigue and imprinting. The polarization dead layer caused by planar oxygen vacancies is shown to be around 72 Å. Microscopic origin and insight, based on the local Ti-O relative displacements, are provided to understand these interesting phenomena.

## Introduction

Extended vacancies in ferroelectrics (FE) are of substantial importance both fundamentally and technologically. Fundamentally, extended vacancies alter the atom-atom interaction over an extensive space region^1^, and break the delicate balance between the long-range and short-range interactions^2,3^, which may profoundly affect the ferroelectricity, polarization, and other materials properties. Extended vacancies may also alter the rotational instability in FEs^4,5^. Furthermore, different from isolated defects, extended defects allow the defects to interact strongly with each other, which may possibly generate new and interesting phenomena that do not exist in perfect bulks or in bulks with isolated defects. While a majority of previous studies of defect physics in FEs focus on isolated defects^6–13^, extended defects are less understood.

Technologically, extended vacancies are important in understanding the long-standing problems such as polarization fatigue and aging, as well as in improving the performances of FE devices^14–16^. FE materials are widely used in non-volatile random access memories^17^, actuators^18^, and charge capacitors, thanks to the large and reversible spontaneous polarization, anomalous Born effective charges^19,20^, ultrahigh electromechanical response^21–23^, interesting morphotropic phase boundary^24–26^, large dielectric response^27,28^, and unusual phase transitions^29–31^. FE solids also exhibit interesting new phenomenon such as avalanche criticality in switching^32^, and promise new application in neuromorphic computation^33^. However, these FE applications could be considerably impaired by the existence of extended defects. For instance, defects are linked to polarization fatigue (which deteriorates the performance of FE devices) and aging (which reduces the lifetime of devices) in polarization switching^34–38^. Oxygen vacancies were also reported to pin the domain wall^39^, which may cause the imprinting of electric polarization.

Interestingly, although vacancies often hamper the performance of FE devices, they can nevertheless be technologically useful by producing properties that are not available in perfect FEs. For example, oxygen vacancies in $\hbox {SrTiO}_{3}$ can generate at low temperature a strong electrical conductivity with carrier mobility as high as 10$^{4}$ cm$^{2}$ V$^{-1}$ s$^{-1}$, when conductivity is desirable^40^. Furthermore, a Pb-O vacancy pair in $\hbox {PbTiO}_3$ was shown to enhance the local polarization by nearly 100%.^41^ Meanwhile, Pb vacancies in Pb($\hbox {Sc}_{0.5}$Nb$_{0.5}$)O$_3$ can broaden the peak of dielectric response^42^. Moreover, vacancies were reported to be able to enhance significantly the electromechanical response in aged $\hbox {BaTiO}_3$.^43^ Since vacancies can be either detrimental or beneficial, it is thus technologically important to find out how to eliminate, or how to (intentionally) generate, vacancies in FEs, depending on whether we want to diminish the detrimental effects or to enhance the beneficial effects.

Despite the importance, extended vacancies in FEs are poorly understood. Many questions of fundamental relevance remain to be answered. These include: (i) Whether are the extended vacancies thermodynamically stable from the fundamental viewpoint of energy? How much is the formation energy for extended vacancies? (ii) Under what conditions (in terms of varying the chemical potentials of the atomic reservoir and the electron reservoir) can the extended vacancies be generated or annihilated, by which we can enhance the beneficial properties or eliminate the harmful effects? (iii) How are ferroelectricity and polarization affected by the extended vacancies? (iv) Extended vacancies may appear in different charge states. How does the impact of extended vacancies on structural properties and ferroelectric polarization depend on the charge states of vacancies?

**Figure 1:**
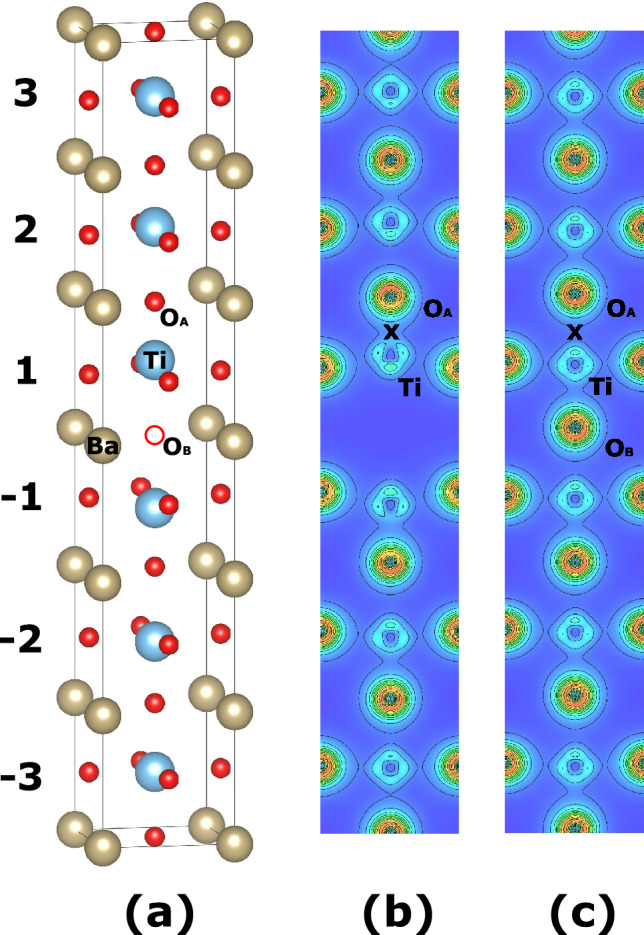
(Color online) (**a**) Three-dimensional view of the planar vacancies in an $1 \times 1 \times 6$ supercell, where an oxygen vacancy is created by removing the $\hbox {O}_{{\textrm{B}}}$ atom. Individual *bulk* cells along the *c*-axis are marked as $-3$, $-2$, $-1$, 1, 2, and 3, depending on their distances from the vacancy plane. (**b**) Charge density contour on the *xz* plane which passes the Ti and $\hbox {O}_{{\textrm{A}}}$ atoms, for $\hbox {BaTiO}_3$ with planar ${\textrm{V}}^{2+}_{{\textrm{O}}}$ vacancies; (**c**) Same as (**b**), but for perfect $\hbox {BaTiO}_3$. In (**c**), the polarization points downward, and the Ti atom moves toward atom $\hbox {O}_{{\textrm{B}}}$. At location X, there is hybridization between Ti and $\hbox {O}_{{\textrm{A}}}$ in (**b**), but not in (**c**).

In this paper we investigate by first-principles density functional theory the extended planar oxygen vacancies in ferroelectric $\hbox {BaTiO}_3$ (BTO), where oxygen vacancies are formed on an atomic plane. $\hbox {BaTiO}_3$ is a prototypical FE solid and is commonly used in FE devices. $\hbox {BaTiO}_3$ with planar oxygen vacancies to be investigated here is illustrated in Fig. 1a, where an oxygen vacancy is created at the center of an $1 \times 1 \times 6$ supercell by removing the oxygen atom $\hbox {O}_{{\textrm{B}}}$. Due to the periodicity along the lateral *ab*-plane directions, the oxygen vacancies thus created are extended on an atomic plane, and will interact strongly with each other. Therefore, the object of our study is essentially an oxygen-depleted plane. We consider the vacancy model in Fig. 1a since it is energetically favorable (see the “Methods” section for detail). Obviously our study differs from the previous work^6–13^ where isolated defects were reported. Extended planar vacancies are potentially more interesting.

The purpose of this study is to determine how much energy is needed to form the planar oxygen vacancies in FE $\hbox {BaTiO}_3$, and to understand how the planar vacancies affect ferroelectricity. We obtain the following main results: (i) The formation energy $\Delta H$ of planar ${\textrm{V}}^{2+}_{{\textrm{O}}}$ vacancies can be interestingly small (on the order of merely 0.54 eV) even under the oxygen-rich condition, showing that planar oxygen vacancies are not as difficult to form as commonly thought. (ii) We systematically determine the optimal condition to eliminate (or generate) the planar ${\textrm{V}}^{q}_{{\textrm{O}}}$ vacancies of all three charge states, which is technologically relevant in terms of growing high-quality $\hbox {BaTiO}_3$. (iii) Planar ${\textrm{V}}^{2+}_{{\textrm{O}}}$ vacancies drastically reduce the ferroelectricity in $\hbox {BaTiO}_3$, which provides a pivotal (theoretical) evidence that the planar oxygen vacancies could be the origin of causing polarization fatigue and imprinting. (iv) The length of polarization dead layer caused by planar ${\textrm{V}}^{2+}_{{\textrm{O}}}$ vacancies in $\hbox {BaTiO}_3$ is determined to be large and around 72  Å, showing that extended oxygen vacancies are very harmful to electric polarization.

## Results and discussions

### Vacancy formation energies and charge states

Whether or not planar ${\textrm{V}}^{q}_{{\textrm{O}}}$ vacancies of a given *q* charge state can occur in $\hbox {BaTiO}_3$ depends on the formation energy $\Delta H$. A lower formation energy indicates that vacancies ${\textrm{V}}^{q}_{{\textrm{O}}}$ are more likely to occur by requiring a less energy. Here it helps to be (slightly) more quantitative. Using a typical oxygen vacancy concentration of 8$\times$10$^{18}$ cm$^{-3}$ which was observed in experiments^44^, it was quantitatively estimated that, when $\Delta H$ is below 1.5 eV, vacancies are very likely to occur^45,46^. When $\Delta H$ is between 1.5 and 4.0 eV, vacancies are still possible to occur, but with less probability. On the other hand, if $\Delta H$ is near or above 4.0 eV, vacancies will be difficult to form.

**Figure 2:**
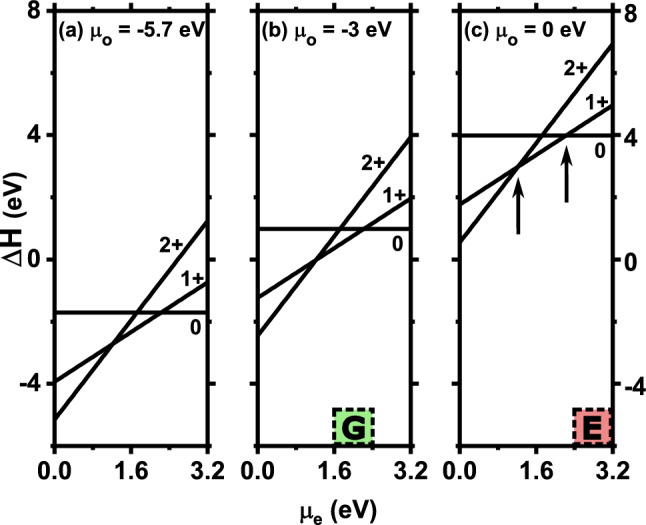
Vacancy formation energy $\Delta H$ as a function of the chemical potential $\mu _{{\textrm{e}}}$ of the electron reservoir, at the following chemical potential $\mu _{{\textrm{O}}}$ of the oxygen reservoir: (**a**) $\mu _{{\textrm{O}}}=-5.7$ eV (oxygen poor), (**b**) $\mu _{{\textrm{O}}}=-3.0$ eV, and (**c**) $\mu _{{\textrm{O}}}=0$ eV (oxygen rich). Charge state *q* of vacancy is labelled near the corresponding line. Shadow box G in (**b**) marks the Condition G, while shadow box E in (**c**) marks the Condition E. The transitions of the vacancy charge states are marked by two vertical arrows in (**c**).

The calculated formation energies of planar ${\textrm{V}}^{q}_{{\textrm{O}}}$ vacancies are shown in Fig. 2a–c as a function of the chemical potential $\mu _{{\textrm{e}}}$ of the electron reservoir, for three different chemical potentials $\mu _{{\textrm{O}}}$ of the oxygen reservoir. We use the experimental band gap of 3.2 eV as the varying range of $\mu _{{\textrm{e}}}$. One possible method to control $\mu _{{\textrm{e}}}$ is by using the gate voltage, in which the Fermi energy of the electron reservoir (such as in two-dimensional electron gas) is altered by applying voltage. Note that $\Delta H$ depends on both $\mu _{{\textrm{e}}}$ and $\mu _{{\textrm{O}}}$, as described in Eq. (1) of the “Methods” section. Figure 2 contains various knowledge about the energetics and stabilities of planar ${\textrm{V}}^{q}_{{\textrm{O}}}$ vacancies. Several key observations can be made from Fig. 2:

First, we see from Fig. 2a that, under the oxygen-poor condition (i.e., when $\mu _{{\textrm{O}}}$ is at its low limit $\mu _{{\textrm{O}}}$ = $-5.7$ eV), $\Delta H$s of the three charge states (${\textrm{V}}^{2+}_{{\textrm{O}}}$, ${\textrm{V}}^{1+}_{{\textrm{O}}}$, and ${\textrm{V}}^{0}_{{\textrm{O}}}$) are largely negative, which indicates that the formation of vacancies requires no energy. The planar oxygen vacancies of all three charge states will thus spontaneously occur and vacancies will be abundant. This is consistent with our intuition: since there are insufficient oxygen atoms in $\hbox {BaTiO}_3$ under the oxygen-poor condition, oxygen vacancies will thus be amply present.

Next let us turn attention to the oxygen-rich condition in Fig. 2c. Interestingly, we discover that planar ${\textrm{V}}^{2+}_{{\textrm{O}}}$ vacancies may occur even under the oxygen-rich condition. More specifically, Fig. 2c reveals that, when $\mu _e$ is close to 0, $\Delta H$ of planar ${\textrm{V}}^{2+}_{{\textrm{O}}}$ vacancies is merely 0.54 eV, and formation of this charge-state vacancies thus requires only a small amount of energy, indicating that extended ${\textrm{V}}^{2+}_{{\textrm{O}}}$ vacancies may easily form under the oxygen-rich condition. This is rather surprising since, according to the common wisdom, oxygen vacancies should be difficult to occur while oxygen is rich and sufficient. Our result suggests that one need be cautious in applying the common wisdom to the extended oxygen vacancies in FEs. One possible reason for the low formation energy of planar ${\textrm{V}}^{2+}_{{\textrm{O}}}$ vacancies in FEs, despite oxygen is rich, is explained later.

Furthermore, we can determine from Fig. 2c which charge state of vacancy is most stable at a given chemical potential $\mu _{{\textrm{e}}}$. Specifically, when $\mu _{{\textrm{e}}}< 1.22$ eV, ${\textrm{V}}^{2+}_{{\textrm{O}}}$ is most stable since its formation energy is lower than both ${\textrm{V}}^{1+}_{{\textrm{O}}}$ and ${\textrm{V}}^{0}_{{\textrm{O}}}$ in Fig. 2c. When $\mu _{{\textrm{e}}}$ is increased to $1.22< \mu _e < 2.22$ eV (i.e., between the two vertical arrows in Fig. 2c), ${\textrm{V}}^{1+}_{{\textrm{O}}}$ becomes most stable. When $\mu _{{\textrm{e}}}$ is further increased to $\mu _e > 2.22$ eV, neutral ${\textrm{V}}^{0}_{{\textrm{O}}}$ is most stable. We thus see that, by varying $\mu _e$, each of the three charge states can be made most stable for planar oxygen vacancies. Note that the $\mu _{{\textrm{e}}}$ dependence of the most-stable charge state is the same in Fig. 2a,b, regardless of the choice of the chemical potential $\mu _{{\textrm{O}}}$.

Our theoretical results that planar oxygen vacancies can be formed in $\hbox {BaTiO}_3$ with reasonably small formation energies call for experiments to verify. Nowadays, the layer-by-layer epitaxial growth of perovskite oxides, enabled by pulsed laser deposition (PLD)^47^ or molecular beam epitaxy (MBE)^48^, shall (in principle) make it possible to fabricate oxide materials with extended defects. Although no planar oxygen vacancies have been experimentally grown per se, evidence for the existence of extended clusters of oxygen vacancies was reported in perovskite oxides^49,50^, which is to some extent consistent with our theory.

### Condition to generate (or eliminate) planar oxygen vacancies

One intriguing question about planar oxygen vacancies is to find the condition (to be termed as “Condition G”) under which these vacancies can be easily *generated* for all three charge states. The knowledge of this condition may be useful in experiments to deliberately grow $\hbox {BaTiO}_3$ with extended ${\textrm{V}}^{q}_{{\textrm{O}}}$ vacancies.

In Fig. 2b, at $\mu _{{\textrm{O}}}=-$3.0 eV (i.e., as $\mu _{{\textrm{O}}}$ is located in the nearby of the middle point between the oxygen-rich and oxygen-poor conditions), the vacancy formation energies of all three charge states are small and around 0.6 eV, when $\mu _{{\textrm{e}}}$ is in the range from 1.6 to 2.4 eV (i.e., within the shadow box G in Fig. 2b). As a result, planar oxygen vacancies of each of the three charge states may easily occur at the considered $\mu _{{\textrm{O}}}$ and $\mu _{{\textrm{e}}}$. Therefore, the Condition G under which all three charge states of vacancies can be easily generated is $\mu _{{\textrm{O}}}\approx -$3.0 eV and 1.6$\le \mu _{{\textrm{e}}}\le$2.4 eV. Within Condition G, one may change the most-stable charge state by slightly altering $\mu _{{\textrm{e}}}$ as in Fig. 2b. Note that changing the charge state of defects is technologically useful since it can drastically tune the transport properties.

Another question regarding planar oxygen vacancies is to figure out the condition (to be termed “Condition E”) under which all three charge states of planar oxygen vacancies can be easily *eliminated* (i.e., these vacancies are difficult to form), for the purpose of minimizing the detrimental effects of oxygen vacancies. Our study also yields the Condition E. From Fig. 2c, we see that, when $\mu _{{\textrm{O}}}$ = 0 eV and when $\mu _{{\textrm{e}}}$ is between 2.4 and 3.2 eV (i.e., within the shadow box E in Fig. 2c), the formation energies of all three charge states are nearly 4.0 eV or above, suggesting that planar oxygen vacancies are unlikely to occur. Therefore, the Condition E is to grow $\hbox {BaTiO}_3$ under the oxygen-rich condition and at the same time adjust $\mu _{{\textrm{e}}}$ to be near the conduction band minimum. Considering that the extended oxygen vacancies are one key class of defects that may cause the fatigue and aging in polarization switching^15^, the Condition E obtained here could be useful in terms of growing $\hbox {BaTiO}_3$ solids that will improve the performance of FE devices.

We also estimate how our calculation results may be affected if the LDA band gap is corrected. We have attempted the hybrid (such as HSE) density functional calculations to correct the band gap^51^. However, we find that the difficulty in charge self-consistency due to charge sloshing and the difficulty in structural relaxation of many atoms in supercell make the computation time consuming. Since one key purpose of the hybrid functional calculations is to correct the band gap, we thus decide to perform LDA+U calculations, which largely improve the band gap and serve (partially) as an useful alternative to address the gap problem. We choose a U=6eV value for Ti atoms, since this U value produces a direct band gap of 3.15 eV at zone-center $\Gamma$ for perfect bulk $\hbox {BaTiO}_3$, which is comparable to the experimental band gap of 3.20 eV. In contrast, the direct band gap of LDA is only 2.40eV at the $\Gamma$ point. Using the LDA+U method, we find that the formation energies $\Delta H$ are modified, but by a rather small change of 0.30eV, 0.35eV, and 0.55eV for planar ${\textrm{V}}^{2+}_{{\textrm{O}}}$, ${\textrm{V}}^{1+}_{{\textrm{O}}}$, and ${\textrm{V}}^{0}_{{\textrm{O}}}$ vacancies, respectively. As discussed quantitatively in Refs.^45,46^, in order for ${\textrm{V}}^{q}_{{\textrm{O}}}$ vacancies to change their probabilities from “being likely to occur” to “being unlikely to occur”, the formation energy needs to change from 1.5 to 4.0 eV, i.e., by about 2.5 eV. The $\Delta H$ modifications (on the order of 0.35 eV) due to LDA+U calculations are considerably smaller than 2.5 eV. Therefore, the band-gap correction does not qualitatively alter the main conclusions in our study. Furthermore, from the LDA+U calculations, the *relative* change in $\Delta H$ (for instance, the change of $\Delta H$ of planar ${\textrm{V}}^{1+}_{{\textrm{O}}}$ vacancies with respect to planar ${\textrm{V}}^{2+}_{{\textrm{O}}}$ vacancies) is even smaller (only 0.05 eV), which reveals that the transition chemical potentials ($\mu _{{\textrm{e}}}$) between different charge states are nearly unchanged.

### Insulating or conducting nature

Defects can cause an insulating solid to become conducting since these defects may introduce extra mobile electrons or holes. It is thus relevant to determine whether the defected system is insulating or metallic. For defects in FE solids, this question is even more critical because mobile electrons or holes in FEs may effectively screen (and thus alter) the electric polarization, if ferroelectricity is to persist.

**Figure 3:**
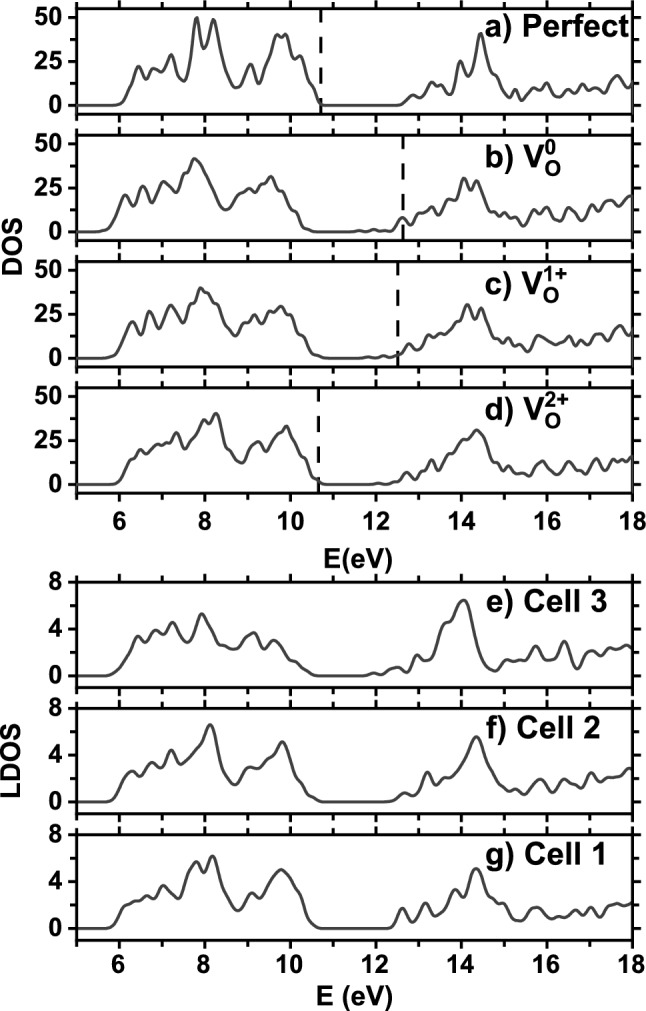
Upper panel: Densities of states (DOS) of (**a**) perfect $\hbox {BaTiO}_3$, (**b**) $\hbox {BaTiO}_3$ with planar ${\textrm{V}}^{0}_{{\textrm{O}}}$ vacancies, (**c**) $\hbox {BaTiO}_3$ with ${\textrm{V}}^{1+}_{{\textrm{O}}}$ vacancies, and (**d**) $\hbox {BaTiO}_3$ with ${\textrm{V}}^{2+}_{{\textrm{O}}}$ vacancies. Dashed line indicates the Fermi level $E_F$ in each system. Lower panel: the projected local DOS in the following bulk cells for $\hbox {BaTiO}_3$ with planar ${\textrm{V}}^{2+}_{{\textrm{O}}}$ vacancies: (**e**) Cell 3, (**f**) Cell 2, and (**g**) Cell 1.

The densities of states (DOS) of $\hbox {BaTiO}_3$ with planar ${\textrm{V}}^{q}_{{\textrm{O}}}$ vacancies are shown in Fig. 3a–d, along with the DOS of perfect $\hbox {BaTiO}_3$. In Fig. 3a–d, the energy levels of different systems are aligned by using the Kohn–Sham orbital energy of Ti semi-core 3*s* state. After alignment, it is physically meaningful to compare the relative orbital energies in different systems if needed, although this comparison is not critical in the present study.

Several conclusions can be made from Fig. 3a–d: (i) For both ${\textrm{V}}^{0}_{{\textrm{O}}}$ and ${\textrm{V}}^{1+}_{{\textrm{O}}}$ planar vacancies, the Fermi level $E_F$ is located within the conduction bands (Fig. 3b,c). These two systems thus have mobile electrons and are conducting. (ii) In contrast, for ${\textrm{V}}^{2+}_{{\textrm{O}}}$ vacancies in Fig. 3d, $E_F$ is located at the top of valence band. $\hbox {BaTiO}_3$ with planar ${\textrm{V}}^{2+}_{{\textrm{O}}}$ vacancies is thus an insulator. (iii) Comparing ${\textrm{V}}^{0}_{{\textrm{O}}}$ in Fig. 3b with ${\textrm{V}}^{1+}_{{\textrm{O}}}$ in Fig. 3c, we see that neutral ${\textrm{V}}^{0}_{{\textrm{O}}}$ has a larger DOS at the Fermi level, and the *q* = 0 charge state may thus enhance the conductivity and/or superconductivity than *q* = 1+. (iv) When *q* increases from Fig. 3b–d, the Fermi level decreases.

The finding of the insulating nature of planar ${\textrm{V}}^{2+}_{{\textrm{O}}}$ vacancies is nontrivial. It reveals that no mobile electrons and holes are available to screen the polarization in $\hbox {BaTiO}_3$ with ${\textrm{V}}^{2+}_{{\textrm{O}}}$. In contrast, the conducting nature of planar ${\textrm{V}}^{0}_{{\textrm{O}}}$ or ${\textrm{V}}^{1+}_{{\textrm{O}}}$ vacancies indicates that polarization will be screened (or at least partially screened).

The theoretical result that $\hbox {BaTiO}_3$ with planar ${\textrm{V}}^{2+}_{{\textrm{O}}}$ vacancies is an insulator can be intuitively understood as follows. In $\hbox {BaTiO}_3$ solid, outside each O atom, there are two additional electrons which are transferred from Ti or Ba cations. These two additional electrons are strongly bonded to the O atom, due to the strong electronegativity of oxygen atoms. When an $\hbox {O}_{{\textrm{B}}}$ atom is removed from $\hbox {BaTiO}_3$ to create a vacancy, the two additional electrons outside $\hbox {O}_{{\textrm{B}}}$ prefer to stay with this atom (rather than staying inside the $\hbox {BaTiO}_3$ solid) in order to lower the energy of the total system. In other words, we need to remove both $\hbox {O}_{{\textrm{B}}}$ and two additional electrons, which leads to that each ${\textrm{V}}^{}_{{\textrm{O}}}$ vacancy carries a 2+ charge. Furthermore, because the two additional electrons outside atom $\hbox {O}_{{\textrm{B}}}$ are removed, these electrons will not be donated to the $\hbox {BaTiO}_3$ solid, and there will be no mobile electrons inside $\hbox {BaTiO}_3$, which makes it an insulator.

While both perfect $\hbox {BaTiO}_3$ (Fig. 3a) and defected $\hbox {BaTiO}_3$ with ${\textrm{V}}^{2+}_{{\textrm{O}}}$ (Fig. 3d) are insulating, their band gaps nevertheless differ. The LDA band gap ($\sim$ 1.3 eV) of defected $\hbox {BaTiO}_3$ with ${\textrm{V}}^{2+}_{{\textrm{O}}}$ vacancies is smaller than that ($\sim$ 2.0 eV) of perfect $\hbox {BaTiO}_3$. A reduction of band gap in FEs could be technologically useful to improve the power conversion efficiency in photovoltaic applications^52,53^.

### Impact on ferroelectricity

One critical subject about extended oxygen vacancies in FEs centers on how they may affect ferroelectricity^1,14^. Here we attempt to shed light on this important topic. Note that the *ab-initio* determination on how charged defects affect ferroelectricity is a standing challenge despite its importance^54,55^, since the polarization itself in *charged* systems is not translation invariant. The determination becomes possible after it was recently proved that the *change* of polarization in charged systems is translation invariant and thus physically meaningful^45^.

From DOS in Fig. 3, we have determined that $\hbox {BaTiO}_3$ with planar ${\textrm{V}}^{2+}_{{\textrm{O}}}$ vacancies is an insulator, and therefore ferroelectricity can, in principle, exist for this charge state. The challenge is to find how large the polarization will be quantitatively. Will the polarization be similar to (or drastically different from) the value in perfect $\hbox {BaTiO}_3$? On the other hand, for planar oxygen vacancies of two other charge states (i.e., ${\textrm{V}}^{0}_{{\textrm{O}}}$ and ${\textrm{V}}^{1+}_{{\textrm{O}}}$), the systems are metallic, and electric polarization is ill-defined for metallic systems^56^.

**Figure 4:**
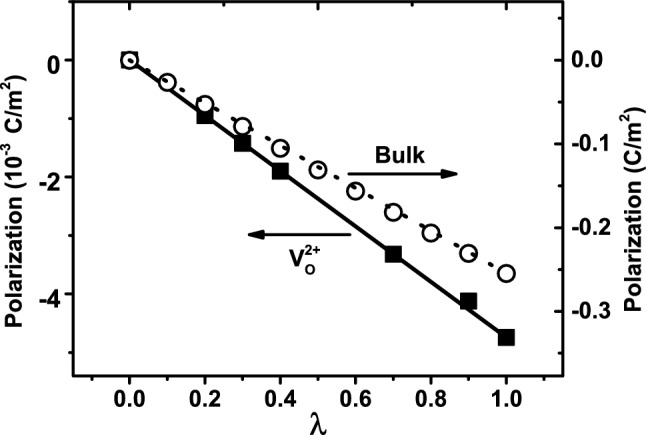
Polarization as a function of the $\lambda$ parameter, for defective $\hbox {BaTiO}_3$ with planar ${\textrm{V}}^{2+}_{{\textrm{O}}}$ vacancies (solid squares and solid line, using the left vertical axis), and for perfect $\hbox {BaTiO}_3$ (empty circles and dotted line, using the right vertical axis). $\lambda$ = 0 corresponds to the centro-symmetric structure, and $\lambda$ = 1 corresponds to the LDA-optimized structure for each system. Note that the left vertical axis is in units of 10$^{-3}$ C/m$^2$. The polarization value at the final configuration $\lambda$ = 1 is determined to be P = $-4.7\times 10^{-3}$ C/m$^2$ for $\hbox {BaTiO}_3$ with ${\textrm{V}}^{2+}_{{\textrm{O}}}$ vacancies, and P = − 0.25 C/m$^2$ for perfect $\hbox {BaTiO}_3$ without vacancies.

We go one step further and perform the Berry-phase calculations to determine the electric polarizations in defective $\hbox {BaTiO}_3$ with planar ${\textrm{V}}^{2+}_{{\textrm{O}}}$ vacancies as well as in perfect bulk, using the modern theory of polarization^54,55^ and the intermediate configurations as described in the “Methods” section. Figure 4 depicts the polarization as a function of the $\lambda$ parameter which controls the intermediate configurations.

For $\hbox {BaTiO}_3$ with planar ${\textrm{V}}^{2+}_{{\textrm{O}}}$ vacancies (see the solid-square symbols and solid line in Fig. 4), we observe the following. (i) The polarization value at $\lambda$ = 0 is zero, showing that this configuration is indeed centro-symmetric and can thus serve as the zero-reference point for determining the change in polarization, as it should be. (ii) The polarization at $\lambda$ = 1 is found to be P = $-4.7\times$ 10 $^{-3}$ C/m$^2$, which is the polarization value for the optimized structure of $\hbox {BaTiO}_3$ with planar ${\textrm{V}}^{2+}_{{\textrm{O}}}$ vacancies.

Meanwhile, for perfect bulk $\hbox {BaTiO}_3$ (see the empty circles and dotted line in Fig. 4), we find that the polarization is P = − 0.25 C/m$^2$ at $\lambda$ = 1. This theoretical value of polarization agrees well with the experimental measurement results^57,58^ of $\vert {\textrm{P}} \vert$ = 0.26 C/m$^2$, indicating that our polarization calculations are rather reliable.

The results in Fig. 4 reveal an important conclusion, that is, the electric polarization in $\hbox {BaTiO}_3$ with planar ${\textrm{V}}^{2+}_{{\textrm{O}}}$ vacancies is more than 50 times smaller than the value in perfect bulk. In other words, we find that planar ${\textrm{V}}^{2+}_{{\textrm{O}}}$ vacancies almost entirely eliminate the ferroelectric polarization, showing that the extended planar oxygen vacancies in $\hbox {BaTiO}_3$ generate a devastating effect on ferroelectricity.

The calculation results also reveal a drastic difference between *extended* oxygen vacancies and *isolated* oxygen vacancies. A previous study has reported that strong ferroelectricity with a polarization of 0.24 C/m$^2$ persists in $\hbox {BaTiO}_3$ with *isolated* ${\textrm{V}}^{2+}_{{\textrm{O}}}$ vacancies^45,46^, which is close to the bulk value of 0.25 C/m$^2$. In sharp contrast, for *extended* planar ${\textrm{V}}^{2+}_{{\textrm{O}}}$ vacancies, we find in Fig. 4 that the polarization nearly vanishes. Therefore, the two types of oxygen vacancies produce very different effects on macroscopic polarization.

Moreover, our studies have significant implication on polarization fatigue. First, our current results confirm that planar oxygen vacancies indeed can give rise to polarization fatigue in FEs by diminishing the polarization, which supports the phenomenological model proposed in Ref.^15^. Second, our results suggest that polarization fatigue in switching is likely caused by extended vacancies (namely, not isolated vacancies), since it is the former that drastically reduces the polarization.

### Origin of the vanished polarization and microscopic insight

**Figure 5:**
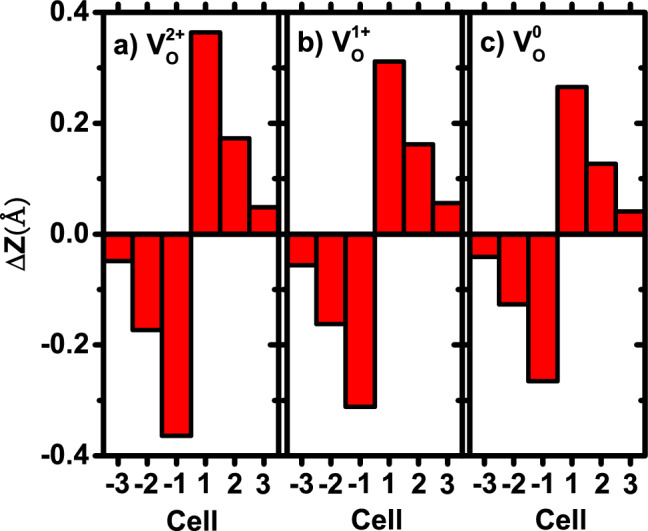
(Color online) Ti-O relative displacements $\Delta Z$ in individual bulk cells (as labelled by the horizontal axis) of the 1$\times$1$\times$6 supercell, for the optimized atomic configurations of the following planar vacancies: (**a**) ${\textrm{V}}^{2+}_{{\textrm{O}}}$, (**b**) ${\textrm{V}}^{1+}_{{\textrm{O}}}$, and (**c**) ${\textrm{V}}^{0}_{{\textrm{O}}}$.

The drastic reduction of polarization caused by planar ${\textrm{V}}^{2+}_{{\textrm{O}}}$ vacancies is interesting and may explain the important phenomenon such as polarization fatigue. We next attempt to provide microscopic insight in order to understand the origin of the declining polarization. For this purpose, we investigate the Ti-O relative displacement $\Delta Z$ along the tetragonal *c*-axis direction, for each $\hbox {TiO}_2$ layer in the LDA-optimized structures of defected $\hbox {BaTiO}_3$ with ${\textrm{V}}^{q}_{{\textrm{O}}}$ vacancies. $\Delta Z$ is defined as $\Delta Z= Z_{{\textrm{Ti}}} - Z_{{\textrm{O}}}$, where $Z_{{\textrm{Ti}}}$ and $Z_{{\textrm{O}}}$ are the *z*-component coordinates of the Ti atom and the base O atoms, respectively. $\Delta Z$ is an indicator of local polarization. Another (slightly different) definition of local polarization is to define $\Delta Z$ as the *z*-coordinate difference between Ti and the center of mass of O atoms. We find that the calculation results of these two $\Delta Z$ quantities are very similar. Since each supercell of vacancy calculations consists of six bulk cells and we need to address $\Delta Z$ in each bulk cell, we thus label, as in Fig. 1a, the six individual bulk cells as $-3$, $-2$, $-1$, 1, 2, 3 based on their distances from the vacancy plane. The obtained $\Delta Z$ displacements are shown in Fig. 5a–c for ${\textrm{V}}^{2+}_{{\textrm{O}}}$, ${\textrm{V}}^{1+}_{{\textrm{O}}}$, and ${\textrm{V}}^{0}_{{\textrm{O}}}$, respectively. Let us first focus on ${\textrm{V}}^{2+}_{{\textrm{O}}}$ in Fig. 5a.

Several important findings can be obtained in Fig. 5a: (i)$\Delta Z$ in cell 1 is found remarkably large, with a magnitude $\Delta Z$ = 0.36 Å. To appreciate the size of this $\Delta Z$, we compare it with the Ti-O displacement in *perfect* bulk $\hbox {BaTiO}_3$ with P4mm symmetry, which is 0.10 Å according to our calculation. $\Delta Z$ in cell 1 for planar ${\textrm{V}}^{2+}_{{\textrm{O}}}$ vacancies is thus $\sim$360% times of the counterpart in perfect $\hbox {BaTiO}_3$. This reveals that the local dipole moment is strongly enhanced in cell 1 near the planar vacancies, supporting the result of Ref.^41^. (ii) $\Delta Z$ decreases drastically from cell 1 to cell 3, being 0.17 Å in cell 2 and 0.05 Å in cell 3. It shows that the impact of planar vacancies declines when cells move away from the vacancies, as a result of the strong dielectric screening in FEs^59–61^. (iii) Interestingly, $\Delta Z$ of cell 1 and $\Delta Z$ of cell $-1$ in Fig. 5a have an equal magnitude, but opposite signs, showing that the strong local dipole moments induced by planar vacancies in cells 1 and $-1$ are cancelled. This explains why the total polarization in Fig. 4 nearly vanishes for ${\textrm{V}}^{2+}_{{\textrm{O}}}$.

Additional microscopic insight can be gained by contrasting the $\Delta Z$ displacements of different charge states in Fig. 5. Two key outcomes can be drawn. First, when charge state *q* decreases from Fig. 5a–c, $\Delta Z$ in cell 1 decreases notably. Quantitatively, $\Delta Z$ in cell 1 is 0.36 Å, 0.31 Å, and 0.27 Å for *q* = 2+, 1+, and 0, respectively. Second, $\Delta Z$ in cell 1 for neutral ${\textrm{V}}^{0}_{{\textrm{O}}}$ (Fig. 5c) nevertheless remains to be much larger than the $\Delta Z$ value (0.10Å) in perfect bulk $\hbox {BaTiO}_3$. Moreover, for all three charge states in Fig. 5, $\Delta Z$ in cell 1 and $\Delta Z$ in cell $-1$ exhibit a mirror symmetry by having opposite signs.

To shed light on why centrosymmetry is restored by planar oxygen vacancies, we examine and depict the charge density of defected $\hbox {BaTiO}_3$ with planar ${\textrm{V}}^{2+}_{{\textrm{O}}}$ vacancies in Fig. 1b and the charge density of perfect $\hbox {BaTiO}_3$ in Fig. 1c, for occupied states in the energy range from 6eV to 11eV of Fig. 3a–d. For perfect $\hbox {BaTiO}_3$ in Fig. 1c, the polarization points downward and Ti moves toward $\hbox {O}_{{\textrm{B}}}$. In contrast, we see in Fig. 1b that, after atom $\hbox {O}_{{\textrm{B}}}$ is removed, the Ti-$\hbox {O}_{{\textrm{B}}}$ bond in the O-Ti-O chain along the *c*-axis is broken. As a consequence, the Ti atom near the vacancy moves toward atom $\hbox {O}_{{\textrm{A}}}$, and a strong hybridization is formed at location X between Ti and $\hbox {O}_{{\textrm{A}}}$ in Fig. 1b, which lowers the energy. This Ti movement considerably weakens the FE polarization by making the structure nearly centrosymmetric. It also gives rise to a large $\Delta Z$ in cell 1 for planar ${\textrm{V}}^{2+}_{{\textrm{O}}}$ vacancies.

We further compute the projected local density of states (LDOS) in different bulk cells for $\hbox {BaTiO}_3$ with planar ${\textrm{V}}^{2+}_{{\textrm{O}}}$ vacancies, and the result is given in Fig. 3e–g. Figure 3g reveals that the strong hybridization between Ti and $\hbox {O}_{{\textrm{A}}}$ leads to a noticeably larger band gap in cell 1 (as compared to cells 2 and 3), which is consistent with the knowledge that stronger hybridization generally gives rise to a larger band gap. Also interestingly, Fig. 3e reveals that the local band gap in cell 3 differs from the gap of perfect bulk, implying that the local structure and local interaction in cell 3 is not yet approaching the perfect bulk. Combining the results in Fig. 1b,c and in Fig. 3e–g, we thus see that the strong (back-bond) hybridization between Ti and $\hbox {O}_{{\textrm{A}}}$ lowers the energy, and meanwhile, works against the ferroelectricity, which is the main reason for the decreasing polarization.

One may also wonder what causes $\Delta Z$ in cell 1 to decline from Fig. 5a–c as charge state *q* decreases. We find that it may be answered by considering the Coulomb interaction. For *positively* charged vacancies such as ${\textrm{V}}^{2+}_{{\textrm{O}}}$ or ${\textrm{V}}^{1+}_{{\textrm{O}}}$, the vacancies will repel Ti cations and attract O anions due to the Coulomb interaction. As a result, Ti$^{4+}$ ions will move away from the vacancy plane, while O$^{2-}$ ions will move toward the vacancy plane, leading to that $\Delta Z$ in cell 1 is enhanced in ${\textrm{V}}^{2+}_{{\textrm{O}}}$ or ${\textrm{V}}^{1+}_{{\textrm{O}}}$ than in neutral ${\textrm{V}}^{0}_{{\textrm{O}}}$.

The above microscopic insight on $\Delta Z$ may also explain why planar ${\textrm{V}}^{2+}_{{\textrm{O}}}$ vacancies can occur with a low formation energy even under the oxygen-rich condition (as seen in Fig. 2c when $\mu _{{\textrm{e}}}$ is near 0). For ${\textrm{V}}^{2+}_{{\textrm{O}}}$, notice that $\Delta Z$ in cell 1 is contributed by both the broken Ti-O bond mechanism (since ${\textrm{V}}^{2+}_{{\textrm{O}}}$ breaks the Ti-$\hbox {O}_{{\textrm{B}}}$ bond) and the Coulomb repulsion mechanism (since ${\textrm{V}}^{2+}_{{\textrm{O}}}$ is positively charged); the combination of these two mechanisms leads to a gigantic $\Delta Z=0.36$ Å in cell 1. This large $\Delta Z$ considerably shortens the distance between Ti and $\hbox {O}_{{\textrm{A}}}$ atoms and allows these two atoms to interact strongly, which stabilizes the planar ${\textrm{V}}^{2+}_{{\textrm{O}}}$ vacancies and lowers the formation energy.

## Polarization dead layer of planar ${\textrm{V}}^{2+}_{{\textrm{O}}}$ vacancies

Denote the spacing distance between two adjacent oxygen-vacancy planes as *S*. Our calculations so far are obtained for $1\times 1\times 6$ supercell, which corresponds to the experimental situation where *S* is around 24 Å (Here, to be consistent, the experimental lattice constant of 4 Å is used). One interesting question then arises: what will happen if the spacing *S* is increased? At what spacing, will ferroelectricity appear? Another important topic of technological relevance concerns how much is the length of the polarization dead layer, to be caused by the planar oxygen vacancies. To address these important questions, we focus on the planar ${\textrm{V}}^{2+}_{{\textrm{O}}}$ vacancies (since, only for ${\textrm{V}}^{2+}_{{\textrm{O}}}$, electric polarization is well defined), and we perform additional calculations using $1\times 1\times 12$, $1\times 1\times 18$, and $1\times 1\times 24$ supercells, which correspond to the experimental situations with spacing *S* being 48 Å, 72 Å, and 96 Å, respectively.

**Figure 6:**
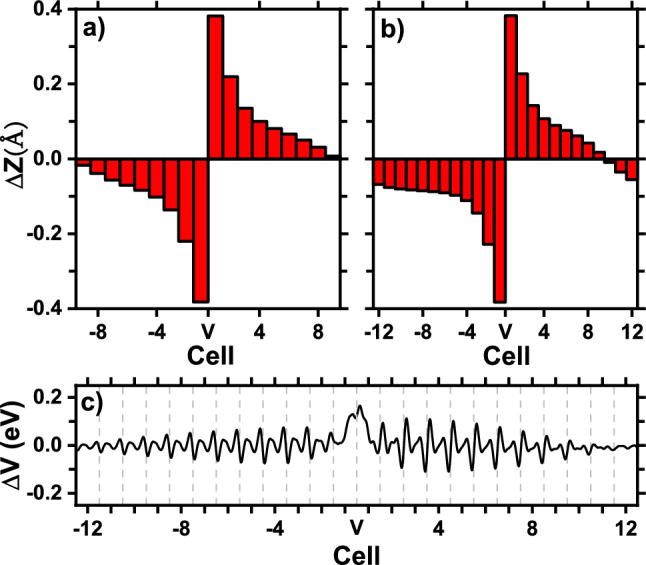
(Color online) Upper panel: Ti-O relative displacements $\Delta Z$ in individual bulk cells (as labelled by the horizontal axis) in $\hbox {BaTiO}_3$ with planar ${\textrm{V}}^{2+}_{{\textrm{O}}}$ vacancies for the following supercells: (**a**) $1\times 1\times 18$; (**b**) $1\times 1\times 24$ supercells. Lower panel: (**c**) average potential difference $\overline{\Delta V}$ between $\hbox {BaTiO}_3$ with planar ${\textrm{V}}^{2+}_{{\textrm{O}}}$ vacancies and perfect FE $\hbox {BaTiO}_3$ for the $1\times 1\times 24$ supercell. V on the horizontal axis indicates the location of the oxygen-vacancy plane.

The obtained Ti-O $\Delta Z$ displacements are given in Fig. 6a,b for the $1\times 1\times 18$ and $1\times 1\times 24$ supercells, respectively. Figure 6a reveals that, when *S* is increased to 72 Å, $\Delta Z$ continues to be centro-symmetric, being positive at one side of vacancy plane and negative at the other side. Polarization remains to be vanishing. Furthermore, the magnitude of $\Delta Z$ in cell 1 (0.38 Å) in Fig. 6a is nearly the same as in Fig. 5a, showing that the *local* structure near the vacancy plane is very similar and does not depend on the spacing distance *S*.

However, and interestingly, when spacing *S* is increased to 96 Å, $\Delta Z$ in Fig. 6b becomes notably different. Specifically, we see in Fig. 6b that (i) while $\Delta Z$ in the left side of vacancy plane is all negative, $\Delta Z$ in the right side of vacancy plane is no longer all positive, and instead it changes from positive in cell 1 to negative in cell 12. As a result, $\Delta Z$ ceases to be centro-symmetric, which is an indication that ferroelectricity emerges in Fig. 6b. (ii) Notice that both $\Delta Z$ in the left boundary (cell $-12$) and $\Delta Z$ in the right boundary (cell 12) in Fig. 6b are negative and point at the same direction. Therefore, the local polarizations at two sides of the oxygen-vacancy plane do not cancel. (iii) The magnitude of $\Delta Z$ in cell $-12$ of Fig. 6b is considerably large (0.068 Å), and is in fact comparable to $\Delta Z$ (0.10 Å) in perfect $\hbox {BaTiO}_3$.

Furthermore, we calculate the electric polarization for the $1\times 1\times 24$ supercell, and the value is found to be $-0.038$ C/m$^2$, which is nonzero. This confirms that ferroelectricity indeed emerges when spacing *S* is 96 Å. Not surprisingly, the above polarization value is small as compared to the value ($-0.25$ C/m$^2$) in perfect $\hbox {BaTiO}_3$, since the *local* polarizations in a large number of bulk cells cancel in Fig. 6b, except those bulk cells far away from the vacancy plane.

Our calculations also answer another important question, namely the length of the polarization dead layer to be caused by the oxygen-vacancy plane. We see from Fig. 6a (with assistance from Fig. 6b) that the length of dead layer is approximately 9 cells (or 36 Å) on each side of the vacancy plane. The (total) length of polarization dead layer is thus 72 Å and is rather large, indicating that planar oxygen vacancies are indeed very harmful to electric polarization. To obtain further insight on how the effect of planar vacancies penetrate into the bulk interior, we calculate the potential difference $\Delta V(x,y,z)$ between defected $\hbox {BaTiO}_3$ with planar ${\textrm{V}}^{2+}_{{\textrm{O}}}$ vacancies and perfect FE bulk $\hbox {BaTiO}_3$, and we then perform the first average over the lateral *xy* directions and perform the second average along *z* direction over the length of one unit cell. The obtained averaged potential difference $\overline{\Delta V}$ is shown in Fig. 6c. By examining the envelope of the potential peaks in different cells on the positive side, we see in Fig. 6c that $\overline{\Delta V}$ decays to zero approximately at cell 9. A similar conclusion can be obtained from the cells on the negative side. This decay length of potential is consistent with the ferroelectric dead layer of 72 Å.

## Conclusions

Planar oxygen vacancies in FE $\hbox {BaTiO}_3$ are investigated by density functional theory and modern theory of polarization. Our main findings are summarized in the following.

(i) Under the oxygen-poor condition, planar ${\textrm{V}}^{q}_{{\textrm{O}}}$ vacancies can spontaneously form and thus abundantly exist in $\hbox {BaTiO}_3$ (Fig. 2a). Interestingly, we also find that, even under the oxygen-rich condition, $\Delta H$ of planar ${\textrm{V}}^{2+}_{{\textrm{O}}}$ vacancies is small and merely 0.54 eV when $\mu _{{\textrm{e}}}$ is near VBM (Fig.2c), and planar ${\textrm{V}}^{2+}_{{\textrm{O}}}$ vacancies are thus very likely to occur. Furthermore, by varying the chemical potential $\mu _{{\textrm{e}}}$, each of the three charge states of oxygen vacancies can be made most stable. According to our calculations in Fig. 2c, ${\textrm{V}}^{2+}_{{\textrm{O}}}$, ${\textrm{V}}^{1+}_{{\textrm{O}}}$, and ${\textrm{V}}^{0}_{{\textrm{O}}}$ are most stable in the regions $\mu _{{\textrm{e}}}< 1.22$ eV, $1.22<\mu _{{\textrm{e}}}< 2.22$ eV, and $\mu _{{\textrm{e}}}>$ 2.22 eV, respectively.

(ii) We determine the optimal condition to grow (or eliminate) the planar oxygen vacancies of all three charge states in $\hbox {BaTiO}_3$. If planar ${\textrm{V}}^{q}_{{\textrm{O}}}$ vacancies are beneficial and need be generated on purpose, the optimal condition (i.e., Condition G) is to choose $\mu _{{\textrm{O}}}$ around $\mu _{{\textrm{O}}}=-$3.0 eV while adjusting $\mu _{{\textrm{e}}}$ to be in the range from 1.6 to 2.4 eV (Fig. 2b), where $\Delta H$s of all three charge states are rather small and $\sim$0.6 eV. Another advantage of choosing the Condition G is that, by altering $\mu _{{\textrm{e}}}$, one can change the most-stable charge state of planar ${\textrm{V}}^{q}_{{\textrm{O}}}$ vacancies, hence tuning the transport properties.

On the other hand, if planar ${\textrm{V}}^{q}_{{\textrm{O}}}$ vacancies are detrimental and need be eliminated, we find that the optimal condition (i.e., Condition E) to eliminate them is to grow $\hbox {BaTiO}_3$ under the oxygen-rich condition ($\mu _{{\textrm{O}}}$ = 0 eV) while adjusting $\mu _{{\textrm{e}}}$ to be near CBM (Fig. 2c).

(iii) $\hbox {BaTiO}_3$ with planar ${\textrm{V}}^{2+}_{{\textrm{O}}}$ vacancies is an insulator, and therefore no mobile charges are available to screen the polarization. The band gap of $\hbox {BaTiO}_3$ with ${\textrm{V}}^{2+}_{{\textrm{O}}}$ vacancies is reduced as compared to perfect $\hbox {BaTiO}_3$. In contrast, $\hbox {BaTiO}_3$ with planar ${\textrm{V}}^{1+}_{{\textrm{O}}}$ (or ${\textrm{V}}^{0}_{{\textrm{O}}}$) vacancies is metallic, and ferroelectricity will be partially screened by mobile charges.

(iv) We quantitatively calculate the electric polarization of defected $\hbox {BaTiO}_3$ with planar ${\textrm{V}}^{2+}_{{\textrm{O}}}$ vacancies, and we find that planar ${\textrm{V}}^{2+}_{{\textrm{O}}}$ vacancies almost completely eliminate the polarization when spacing *S* is 24 Å, showing that planar oxygen vacancies have a devastating effect on ferroelectricity. Our calculations also provide a rather strong evidence that polarization fatigue is likely caused by extended defects rather than isolated defects.

(v) By examining the Ti-O $\Delta Z$ displacement, we find that, for planar ${\textrm{V}}^{q}_{{\textrm{O}}}$ vacancies of all three charge states, $\Delta Z$ in cell 1 is much enhanced by $\sim$300% than in perfect $\hbox {BaTiO}_3$ (Fig. 5). The $\Delta Z$ enhancement in cell 1 mainly originates from the broken Ti-O bond when vacancies are created. Furthermore, $\Delta Z$ in cell 1 declines when charge state *q* changes from +2 to 0, due to the decreasing Coulomb interaction. Moreover, when spacing *S* is 24 Å, $\Delta Z$ is found to exhibit a mirror symmetry for all three charge states, leading to the fact that polarization is largely eliminated by the planar vacancies.

(vi) When spacing *S* is increased to 96 Å, we find that ferroelectricity emerges, as demonstrated by the local Ti-O displacements in the region far away from the vacancy plane (Fig. 6b). The total length of polarization dead layer caused by planar ${\textrm{V}}^{2+}_{{\textrm{O}}}$ vacancies is shown to be around 72 Å.

Considering that extended vacancies profoundly alter the ferroelectric properties and may have important (technological) implications on polarization fatigue and imprinting, we thus hope this study will stimulate more theoretical and experimental interest in the extended vacancies in ferroelectrics.

## Methods

### Determination of vacancy formation energy

Oxygen vacancies in $\hbox {BaTiO}_3$ can exist in three different charge states—${\textrm{V}}^{0}_{{\textrm{O}}}$, ${\textrm{V}}^{1+}_{{\textrm{O}}}$, and ${\textrm{V}}^{2+}_{{\textrm{O}}}$. These vacancies will be denoted in general as ${\textrm{V}}^{q}_{{\textrm{O}}}$, where *q* is the charge state of the vacancy. The formation energy of an oxygen vacancy, $\Delta H$, is defined as the energy that is needed to move an oxygen atom from a perfect $\hbox {BaTiO}_3$ solid to the *atomic* reservoir of oxygen. If the vacancy carries $+q$ charge, $\Delta H$ also includes the energy that is needed to move *q* electrons from the $\hbox {BaTiO}_3$ solid to the *electron* reservoir^62,63^.

Quantitatively, the vacancy formation energy is the energy difference between the initial state ($E_{{\textrm{initial}}}$) and the final state ($E_{{\textrm{final}}}$), i.e., $\Delta H= E_{{\textrm{final}}} -E_{{\textrm{initial}}}$. Here the initial state is the perfect $\hbox {BaTiO}_3$, of which the total energy per supercell is denoted as $E[\mathrm BTO]$. The final state includes three parts^62,63^: (i) The defective $\hbox {BaTiO}_3$ solid containing vacancies ${\textrm{V}}^{q}_{{\textrm{O}}}$, of which the total energy per supercell is denoted as $E[{\textrm{V}}^{q}_{{\textrm{O}}}]$; (ii) Vacated O atoms that are placed in the atomic reservoir of oxygen, where the relative chemical potential of the atomic reservoir is $\mu _{{\textrm{O}}}$ with respect to the reference energy $E_{{\textrm{O}}}$ (Here $E_{{\textrm{O}}}$ is chosen to be the energy of one oxygen atom in an $\hbox {O}_2$ molecule^10,11^); (iii) *q* electrons in the electron reservoir, where the relative chemical potential of the electron reservoir is $\mu _{{\textrm{e}}}$ with respect to the Kohn–Sham orbital energy $\varepsilon '_{{\textrm{VBM}}}$ of the valence band maximum (VBM) of defective $\hbox {BaTiO}_3$ with vacancies. Denote the orbital energy of the VBM of perfect $\hbox {BaTiO}_3$ as $\varepsilon _{{\textrm{VBM}}}$. $\varepsilon '_{{\textrm{VBM}}}$ and $\varepsilon _{{\textrm{VBM}}}$ are related by $\varepsilon '_{\textrm{VBM}} =\varepsilon _{{\textrm{VBM}}} + \Delta {\bar{V}}$, where $\Delta {\bar{V}}$ is the difference in potential far away from the defect. $\Delta {\bar{V}}$ is computed by using the semi-core state of Ti 3*s* orbital^10,11^, since Ti 3*s* state is highly localized with very little energy dispersion—and the difference in the orbital energy of this state in different systems thus reflects $\Delta {\bar{V}}$. By combining the different parts in the above, the formation energy $\Delta H$ per ${\textrm{V}}^{q}_{{\textrm{O}}}$ vacancy is then given by the equation^62,63^

1 $$\begin{aligned} \Delta H = E[{\textrm{V}}^{q}_{{\textrm{O}}}] + (E_{{\textrm{O}}} + \mu _{{\textrm{O}}}) + q(\varepsilon _{{\textrm{VBM}}} + \Delta {\bar{V}} + \mu _e) - E[\mathrm BTO]\,. \end{aligned}$$

Large $\Delta H$ implies that vacancy ${\textrm{V}}^{q}_{{\textrm{O}}}$ is difficult to form by requiring more energy. Note that $\Delta H$ is the vacancy formation energy *per vacancy*, and does not depend on the number of oxygen vacancies. Charge correction is included. Details on how to calculate each term in Eq. (1) were given in Refs.^10,11^.

In Eq. (1), the system to be simulated is to grow the $\hbox {BaTiO}_3$ solid in thermodynamical equilibrium with Ba, Ti, and O atomic reservoirs and with the electron reservoir, to mimic the situation such as MBE or MOCVD growth. During the growth process, atoms in the atomic reservoirs (or electrons in the electron reservoir) can deposit onto, or escape from, the $\hbox {BaTiO}_3$ surface, and oxygen vacancies may form. The chemical potentials of the atomic reservoirs can be changed by varying the pressure and/or temperature of sealed ampoules. The chemical potential of the electron reservoir (electron gas) can be changed either by varying the pressure/temperature of the electron gas or by using gate voltage.

Vacancy formation energy depends on the chemical potential $\mu _{{\textrm{O}}}$ of the oxygen atomic reservoir and the chemical potential $\mu _{{\textrm{e}}}$ of the electron reservoir, as shown in Eq. (1). The allowable ranges of the chemical potentials of atomic reservoirs need be determined using the thermodynamical constraints^62^, so that no (unwanted) secondary phases are formed during the growth of $\hbox {BaTiO}_3$. The details of these constraints for $\hbox {BaTiO}_3$ were described in Refs.^10,11^. Using the thermodynamical constraints, we determine that $\mu _{{\textrm{O}}}$ can vary from −5.7 eV to 0 eV, where the lower limit $\mu _{{\textrm{O}}}=-$5.7 eV corresponds to the oxygen-poor condition for growing $\hbox {BaTiO}_3$ while the upper limit $\mu _{{\textrm{O}}}$=0 eV corresponds to the oxygen-rich condition. The chemical potential $\mu _{{\textrm{e}}}$ of the electron reservoir can vary from the valence band maximum to the conduction band minimum (CBM) of the $\hbox {BaTiO}_3$ solid, as required by the thermodynamical equilibrium between the $\hbox {BaTiO}_3$ solid and the electron reservoir^62^.

Technically we use the first-principles density functional theory within the local density approximation (LDA)^64,65^, as implemented in Quantum Espresso^66,67^, to perform the structural optimization and total energy calculations. Norm-conserving pseudopotentials of Troullier and Martins type are used^68^. The semi-core orbits of Ti 3*s* and 3*p* are treated as valence states to ensure better accuracy; details of pseudopotential generation were given in Ref. ^69^. The energy cutoff for the plane-wave expansion of single-particle Kohn–Sham states is 90 Ry, which is sufficient for the convergence of total energy to 1 meV. We consider tetragonal $\hbox {BaTiO}_3$ with P4mm symmetry. For tetragonal bulk $\hbox {BaTiO}_3$, we obtain the inplane lattice constant *a* = 3.929  Å and the tetragonality *c*/*a* = 1.007, which agree well with the values *a* = 3.945 Å and *c*/*a* = 1.009 in other calculation^70^.

In tetragonal $\hbox {BaTiO}_3$ with P4mm symmetry, there are two inequivalent oxygen Wyckoff sites according to the group theory^71^: one is located on the base plane of an oxygen octahedron, and the other is located at the apex of the octahedron. It was previously shown in the study of isolated defects that the vacancy formation energies at two different sites are very close^10,11^, since the *c*/*a* ratio of tetragonal $\hbox {BaTiO}_3$ is close to 1.0, and the chemical bonds at two oxygen sites are similar. Furthermore, the oxygen vacancies at the base plane do not affect strongly the ferroelectricity and are of less interest. We thus choose to consider the oxygen vacancies at the apical site. We use the $1\times 1 \times 6$ supercell as shown in Fig. 1a. Different charge states (${\textrm{V}}^{0}_{{\textrm{O}}}$, ${\textrm{V}}^{1+}_{{\textrm{O}}}$, and ${\textrm{V}}^{2+}_{{\textrm{O}}}$) of oxygen vacancies are considered in our calculations. A uniform jellium background charge is employed to deal with the charge non-neutrality so that the Coulomb energy does not diverge. For each charge state, the atomic positions and cell parameters are relaxed. A Monkhorst-Pack k-mesh of $6 \times 6 \times 2$ is used in all supercell calculations^72^.

Extended planar oxygen vacancies may include complexes of several closely spaced vacancies in one plane, and many models are thus possible. Here we choose to study an infinite plane with two oxygen vacancies immediately next to each other (i.e., with two vacancies separated by one lattice constant) as in Fig. 1a, and this model will be called the closely-packed configuration, based on the following considerations. (i) It is energetically favorable. For this purpose, we calculate and compare the vacancy formation energy $\Delta H$ for two different oxygen-vacancy configurations: the first one is the closely-packed configuration; the second one is that the oxygen vacancies are separated from each other by two bulk lattice constants, and this model is to be called the unclosely-packed configuration. We find that, under the same chemical potentials of $\mu _{{\textrm{e}}}$ = 0 eV and $\mu _{{\textrm{O}}}$ = 0 eV, $\Delta H$ is 0.54 eV, 1.76 eV, and 3.99 eV respectively for planar ${\textrm{V}}^{2+}_{{\textrm{O}}}$, ${\textrm{V}}^{1+}_{{\textrm{O}}}$, and ${\textrm{V}}^{0}_{{\textrm{O}}}$ vacancies, for the closely-packed configuration. Meanwhile, for the unclosely-packed configuration, $\Delta H$ is 1.09 eV, 3.10 eV, and 5.18 eV for planar ${\textrm{V}}^{2+}_{{\textrm{O}}}$, ${\textrm{V}}^{1+}_{{\textrm{O}}}$, and ${\textrm{V}}^{0}_{{\textrm{O}}}$ vacancies, respectively. Therefore, for each charge state, the closely-packed configuration has a lower energy than the unclosely-packed configuration, showing that oxygen vacancies prefer to stay immediately next to each other. (ii) Considering that extended oxygen defects in FEs are poorly understood, and considering that polarization fatigue and aging is a long-standing problem (while FEs possess many unusual properties^17–33^ such as avalanche criticality in switching^32^ and exciting potential in neuromorphic computation^33^), we hope that the interesting conclusions of the present study, obtained with even not so sophisticated model, are still valuable to the community.

### Determination of electric polarization

Both ions and electrons contribute to electric polarization. Contribution from ions can be calculated straightforwardly using point charges as ${\textbf{P}}_{{\textrm{ion}}}=\frac{1}{\Omega }\sum _i q_i {\textbf{r}}_i$, where $q_i$ is the charge of ion *i* and ${\textbf{r}}_i$ is the position. Contribution from electrons is nontrivial since electrons in solids cannot be treated as point charges, and this contribution need be calculated using the Berry geometric-phase approach, as formulated in the modern theory of polarization (MTP)^54,55^,

2 $$\begin{aligned} {\textbf{P}}_{{\textrm{el}}}=\frac{2e}{(2\pi )^3}\int d{\textbf{k}}_{\perp }\,{\textrm{Im}} \ln \prod _j {\textrm{det}} (\langle u_{m,{\textbf{k}}_{\parallel }^j}|u_{n,{\textbf{k}}_{\parallel }^{j+1}}\rangle ), \end{aligned}$$

where $u_{n{\textbf{k}}}$s are the cell-periodic Bloch part of wave functions for occupied valence states; ${\textbf{k}}_{\perp }$ and ${\textbf{k}}_{\parallel }$ are the components of the electron wave vector perpendicular and parallel to the polarization direction, respectively. Electronic contribution ${\textbf{P}}_{{\textrm{el}}}$ can be further analyzed using the theory of polarization structure^73^. Here one may argue whether MTP is valid for charged systems since the polarization of charged systems is not translation invariant. Recently, Raeliarijaona and Fu showed rigorously that, even for charged systems, the *change* of polarization is still translation invariant and is physically meaningful, and hence the modern theory of polarization remains valid in computing the change of polarization^45^.

We use the following scheme to determine the electric polarization for defected $\hbox {BaTiO}_3$ with vacancies. We first perform structural optimization for $\hbox {BaTiO}_3$ with ${\textrm{V}}^{q}_{{\textrm{O}}}$ vacancies. Denote the optimized atomic configuration as {${\textbf{r}}^{{\textrm{opt}}}_i$}. We then generate a centrosymmetric atomic configuration, {${\textbf{r}}^{{\textrm{c}}}_i$}, of which the polarization is zero by symmetry. The atomic positions of the centrosymmetric phase is constructed as the following. Consider arbitrary atom *i* with *z*-coordinate $Z_i$, and atom *i* will have a corresponding atom (denoted as atom $i'$) if we flip atom *i* using the *xy* plane as the mirror plane. Denote the *z*-coordinate of atom $i'$ as $-Z_{i'}$ (note that $Z_i\ne Z_{i'}$ in general, when the system does not have centrosymmetry). Then, in the *centrosymmetric* phase, the *z*-coordinate positions of atoms *i* and atom $i'$ are constructed to be at $\frac{1}{2}(Z_i+Z_{i'})$ and $-\frac{1}{2}(Z_i+Z_{i'})$ respectively, which are centrosymmetric. Next we adiabatically move atomic positions from {${\textbf{r}}^{{\textrm{c}}}_i$} to {${\textbf{r}}^{\textrm{opt}}_i$} by constructing a series of intermediate configurations according to ${\textbf{r}}_i={\textbf{r}}^{{\textrm{c}}}_i + \lambda ({\textbf{r}}^{\textrm{opt}}_i-{\textbf{r}}^{{\textrm{c}}}_i)$, where $0\le \lambda \le 1$. For each intermediate configuration we compute the electric polarization in order to determine the polarization branch and to ensure the continuity of the computed polarization values along the path. The polarization value at $\lambda =1$ is the needed polarization that corresponds to the optimized configuration of vacancies ${\textrm{V}}^{q}_{{\textrm{O}}}$. This procedure of using intermediate configurations is critical for determining the electric polarization in large *supercells*, since polarization can easily jump discontinuously to other branches of different polarization quanta^54,74^.

## Data Availability

The data that support the findings of this study are available from the corresponding author upon reasonable request.
